# Cardiac Autonomic Modulations and Psychological Correlates in the Yukon Arctic Ultra: The Longest and the Coldest Ultramarathon

**DOI:** 10.3389/fphys.2018.00035

**Published:** 2018-02-12

**Authors:** Lea C. Rundfeldt, Martina A. Maggioni, Robert H. Coker, Hanns-Christian Gunga, Alain Riveros-Rivera, Adriane Schalt, Mathias Steinach

**Affiliations:** ^1^Charité - Universitätsmedizin Berlin, Institute of Physiology, Center for Space Medicine and Extreme Environments Berlin, Berlin, Germany; ^2^Department of Biomedical Sciences for Health, Università degli Studi di Milano, Milan, Italy; ^3^Institute of Arctic Biology, University of Alaska-Fairbanks, Fairbanks, AK, United States; ^4^Department of Physiological Sciences, Pontificia Universidad Javeriana, Bogotá, Colombia

**Keywords:** cold, exercise performance, extreme environments, fatigue, heart rate variability, mood, subarctic ultramarathon, ultra-endurance

## Abstract

Studies on human physical performance in extreme environments have effectively approached the investigation of adaptation mechanisms and their physiological limits. As scientific interest in the interplay between physiological and psychological aspects of performance is growing, we aimed to investigate cardiac autonomic control, by means of heart rate variability, and psychological correlates, in competitors of a subarctic ultramarathon, taking place over a 690 km course (temperatures between +5 and −47°C). At baseline (PRE), after 277 km (D1), 383 km (D2), and post-race (POST, 690 km), heart rate (HR) recordings (supine, 15 min), psychometric measurements (Profile of Mood States/POMS, Borg fatigue, and Karolinska Sleepiness Scale scores both upon arrival and departure) were obtained in 16 competitors (12 men, 4 women, 38.6 ± 9.5 years). As not all participants reached the finish line, comparison of finishers (FIN, *n* = 10) and non-finishers (NON, *n* = 6), allowed differential assessment of performance. Resting HR increased overall significantly at D1 (FIN +15.9; NON +14.0 bpm), due to a significant decrease in parasympathetic drive. This decrease was in FIN only partially recovered toward POST. In FIN only, baseline HR was negatively correlated with mean velocity [*r* −0.63 (P.04)] and parasympathetic drive [pNN50+: *r* −0.67 (P.03)], a lower HR and a higher vagal tone predicting a better performance. Moreover, in FIN, a persistent increase of the long-term self-similarity coefficient, assessed by detrended fluctuation analysis (DFAα2), was retrieved, possibly due to higher alertness. As for psychometrics, at D1, POMS Vigor decreased (FIN: −7.0; NON: −3.8), while Fatigue augmented (FIN: +6.9; NON: +5.0). Sleepiness increased only in NON, while Borg scales did not exhibit changes. Baseline comparison of mood states with normative data for athletes displayed significantly higher positive mood in our athletes. Results show that: the race conditions induced early decreases in parasympathetic drive; the extent of vagal withdrawal, associated to the timing of its recovery, is crucial for success; pre-competition lower resting HR predicts a better performance; psychological profile is reliably depicted by POMS, but not by Borg fatigue scales. Therefore, assessment of heart rate variability and psychological profile may monitor and partly predict performance in long-duration ultramarathon in extreme cold environment.

## Introduction

Human physiology is characterized by continuous reactive adaptation to internal and external conditions and stressors (Ramirez et al., 1999; Hawley et al., 2014). Subjects exposed to extreme conditions and environments display astounding adaptive potential, which ultimately ensures optimal adjustment to current organismic demands and external stress (Kälin et al., 2012; Gunga, 2014). Assessment of autonomic cardiac modulation by means of heart rate variability (HRV) has shown to be a reliable tool to evaluate not only physiological changes (Taralov et al., 2015; Kobayashi et al., 2016), but also psychological aspects of human reactive adaptation to different stressors (Souza et al., 2013). Therefore, HRV assessment may describe human resilience, as it represents a bridge between physiology and psychology, and, by integrating these two aspects, it mirrors human adaptive ability (Thayer et al., 2009; Spangler and Friedman, 2015). Particularly in endurance athletes, training effects, performance level and physical wellbeing may be contextualized through HRV assessment (Atlaoui et al., 2007; Plews et al., 2013a; Buchheit, 2014; Bellenger et al., 2016a). Successful adaptation to increased training load, resulting in improved performance, is associated with increased HRV, as well as enhanced parasympathetic predominance at rest (Plews et al., 2013b; Stanley et al., 2015; Lucini et al., 2017). Assessment of autonomic cardiac modulation conducted directly post-exercise or after competitions, demonstrated a decrease in HRV and a parasympathetic withdrawal (Bricout et al., 2010; Buchheit et al., 2010; Bellenger et al., 2016a), which, however, was effectively recovered depending on the intensity of the preceding exercise (Martinmäki and Rusko, 2008; Manzi et al., 2009; Stanley et al., 2015), and on the individual's training status (Bricout et al., 2010; Buchheit et al., 2010; Bellenger et al., 2016a). This has been vastly evidenced in endurance exercisers (Buchheit et al., 2010; Plews et al., 2012; Da Silva et al., 2014; Kiviniemi et al., 2014), and investigations of cardiac autonomic function in response to extreme endurance exercise, such as ultramarathon, display similar findings (Gratze et al., 2005; Scott et al., 2009; Foulds et al., 2014), even though specific studies related to cardiac autonomic modulation during ultramarathon, are still scarce in comparison. On the other hand, in ultra-endurance athletes, physical exertion has been commonly associated with mental fatigue and increased mood disturbance (Anglem et al., 2008; Siegl et al., 2017), especially in participants who experience adverse incidents, then perform poorly or are forced to prematurely withdraw (Parry et al., 2011; Joslin et al., 2014).

Ultramarathon is mostly defined by course lengths exceeding marathon distance and is characterized by the combination of extremely challenging highly intensive exercise (e.g., track lengths >300 km or great elevation gains), often under strenuous environmental conditions, with concurrently impaired possibilities to recover. The Yukon Arctic Ultra (YAU) is considered to be one of the world's toughest ultramarathons (Coker et al., 2017), as it combines the great course distance of 690 km with extreme environmental conditions typical of a subarctic winter. Except for several in-race checkpoints, there are no indoors sleeping vacancies, so that competitors have to camp on the race-course and experience complete environmental exposition. Therefore, YAU competitors are challenged by a *three-folded stress stimulus* of (i) long-term strenuous exercise, (ii) extreme cold exposure, and (iii) impaired resting conditions, due to in-race camping. So far, among studies on ultra-endurance exercise, research objectives mostly diverge from evaluation of autonomic cardiac function in ultramarathon runners (Degache et al., 2014; Hurdiel et al., 2015; Mrakic-Sposta et al., 2015; Wüthrich et al., 2015; Tonacci et al., 2017), which, to our knowledge, was only implemented in three previous studies (Gratze et al., 2005; Scott et al., 2009; Foulds et al., 2014). However, these investigations differed regarding (i) the race length (e.g., 160 km ultramarathon, or Ironman competition with a total distance of 226.35 km), (ii) study protocol (i.e., pre- vs. post-race comparison only), (iii) environmental conditions (mild climate, summer), and (iv) terrain characteristics (e.g., mountain, large altitude variation, etc.). Therefore, this is the first study to assess cardiac autonomic modulation in competitors of an extremely long (i.e., 690 km) ultramarathon on a mostly flat course, in subarctic climate, which may provide essential insights into the adaptive capacity, as, aside from exercise, HRV is associated with numerous external and internal factors (Rajendra Acharya et al., 2006; Shaffer et al., 2014).

With outdoor temperatures ranging from +5 to −47°C and the air humidity accounting for up to 100%, YAU competitors face extreme subarctic weather conditions. Comparable scientific knowledge is, however, insufficient. Autonomic balance has been observed to shift toward greater parasympathetic predominance during Antarctic stays (Farrace et al., 2003; Harinath et al., 2005), but these results were obtained in expeditioners confined to indoors housing. Moreover, a significant interplay between autonomic cardiac regulation and psychological wellbeing has been observed (Sakuragi et al., 2002; Karavidas et al., 2007; Sgoifo et al., 2015), so that psychometric assessment may also serve to contextualize findings about HRV (Bellenger et al., 2016b; Flatt et al., 2017b). Increased performance and greater parasympathetic drive in cardiac autonomic regulation are associated with increased psychological wellbeing (Cervantes Blásquez et al., 2009; Bisschoff et al., 2016). Conversely, fatigued states and increased mood disturbance have been related to decreased indexes of total HRV, as well as parasympathetic tone (Nuissier et al., 2007; Leti and Bricout, 2013; Schmitt et al., 2013; Flatt et al., 2017a). In this context, impaired resting conditions present another vital influence on cardiac autonomic regulation. Assessment under concurrent sleep-deprivation, which itself is again related to impaired both cognitive and physical performance (Marcora et al., 2009; Fullagar et al., 2015), shows decreased HRV indexes in the parasympathetic domain (Dettoni et al., 2012; Glos et al., 2014; Tobaldini et al., 2014).

To our knowledge, exposition to such a particular combination of stress-stimuli as presented by competition in the YAU has never been investigated regarding cardiac autonomic function and psychological profile. Therefore, we assessed autonomic cardiac regulation in terms of HR/HRV, as well as psychometric measurements including mood states, indicators of sleepiness, exertion and recovery, to investigate adaptation to extreme conditions and performance, by analyzing cardiac autonomic control and its interplay with mood and fatigue. We hypothesized that higher performing competitors, compared to less successful athletes, would exhibit differential profiles of autonomic cardiac regulation associated with optimal psychometric profile, overall characterized by higher adaptability and greater resilience to the extreme challenges of the three-folded stress stimulus.

## Materials and methods

### Subjects and study implementation

This study is part of a larger investigation regarding “*Physiological changes of participants of the Yukon Arctic Ultra - an ultramarathon in extremely cold climate*,” where it is planned to assess a variety of different physiological parameters and their interplay.

From a total number of 78 athletes partaking in the 690 km foot-race category of the YAU during the years 2013, 2015, and 2017, 27 (20 men, 7 women) volunteers enrolled in the study (8 in 2013, 9 in 2015 and 10 in 2017). Due to issues related to data collection, from the 27 participants, only 16 (ALL: 12 men, 4 women) were included in the data analysis (see section Statistics). The majority (*n* = 15) were of Caucasian descent and one was of Asian origin. Their anthropometric data are presented in Table 1.

**Table 1:** Subject demographics.

**Group**	**Gender**	***n***	**Age, years mean (S.D.)**	**Weight, kg mean (S.D.)**	**Height, cm mean (S.D.)**	**BMI, kg/m^2^ mean (S.D.)**
FIN	Men	7	42 (10)	80 (9)	176 (6)	25.7 (3.0)
	Women	3	38 (10)	61 (2)	168 (10)	21.7 (3.4)
	All	10	40 (9)	74 (12)	174 (8)	24.5 (3.5)
NON	Men	5	33 (7)	79 (12)	179 (10)	24.7 (1.7)
	Women	1	51 (0)	58 (0)	170 (0)	20.1 (0)
	All	6	36 (10)	76 (14)	177 (10)	23.9 (2.4)
ALL	Men	12	38 (10)	80 (10)	177 (7)	25.2 (2.5)
	Women	4	41 (11)	60 (2)	169 (9)	21.3 (2.9)
	All	16	39 (10)	75 (12)	175 (8)	24.3 (3.1)

*Subject demographics at baseline for all participants (ALL) and in subgroups (FIN and NON). No significant differences between groups*.

The recruitment for this study was conducted with the support of the event organizers. A call for participants, with a brief description of the study and planned measurements, was transmitted to the athletes who had enrolled in the 690 km foot-race category. The organizers were encouraged to predominantly contact experienced athletes, who had a long history of completed endurance events and/or who had completed the YAU before. Athletes who were interested in the study contacted the principal investigator via e-mail and received further detailed information. The potential study participants had several weeks to ask questions via e-mail and to decide whether to partake in the study or not. There were no further inclusion or exclusion criteria: all athletes enrolled in the 690 km foot-race category were eligible to enter the study. All athletes were required to present to the event organizers a health certificate issued by their home physician, in order to be enrolled in the race. During a meeting in Whitehorse, Yukon Territory, Canada, 4–5 days before the race start, the potential study participants met with the investigators in person, had the chance to ask further questions and to finally give their informed written consent to partake in the study. The study was approved by the Charité Ethics Board (review number EA4/109/12), and all measurements and procedures complied with the Declaration of Helsinki (54th Revision 2008, Korea)^1^ regarding the treatment of human subjects.

All study participants included in the final analysis had completed either one marathon (9.6 ± 24.4) or ultramarathon (14.4 ± 24) prior to their study-participation. The mean longest ultramarathon distance completed by the athletes before their YAU participation was 380 ± 220 km. In addition, seven of the study participants had previously participated in the YAU foot-race in various distance categories, with a mean longest completed distance of 478 ± 219 km. Thus, the study participants were experienced endurance athletes, which is also reflected by their self-reported sedentary HR of 52.6 ± 7.3 bpm. From one participant, this background data was not made available.

### The Yukon arctic ultra: the longest, the coldest ultramarathon

**Figure 1:**
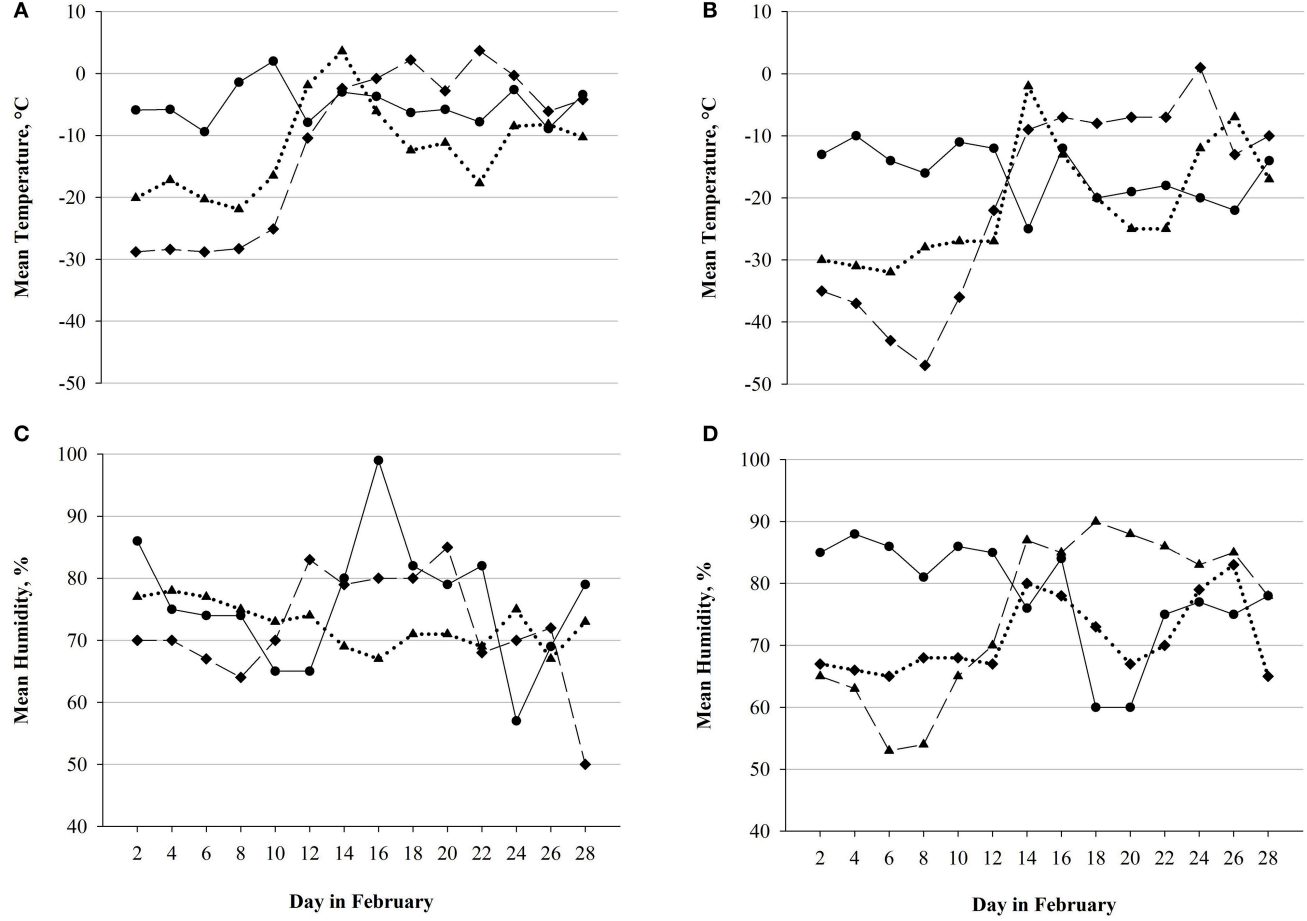
Weather conditions across editions. Mean temperature and mean humidity as measured in Whitehorse and Dawson City in February 2013 (solid line), February 2015 (long dashed line), and February 2017 (dotted line). **(A)** Mean temperature in Whitehorse. **(B)** Mean temperature in Dawson City. **(C)** Mean humidity in Whitehorse. **(D)** Mean humidity in Dawson City. No significant differences between editions or locations^4^.

The Montane® YAU ultra-endurance race takes part in the beginning of February, covering a 690 km distance between Whitehorse and Dawson City in the Canadian Yukon Territory. Besides the foot-race, the YAU also allows the competition in cross-country-skiing and mountain-biking. The first and last sections of the trail account for elevations between 500 and 700 m, however, especially in the last 200 km, the terrain along the Yukon river partly exhibits great elevation gains (up to 1,000 m). The YAU is not an orientation race, as the trail is marked and prepared with snow-mobiles. Via GPS devices, athletes can be tracked on the course and have the possibility to call for assistance in case of an emergency. To further increase their safety, the time until the race has to be completed is limited to 14 days and additionally, medical screenings are administered at the 10 checkpoints which are located (mostly about 50 km apart) along the route. Despite these partly indoor vacancies (otherwise, tents were provided), during the race, competitors face complete exposition to the subarctic environment, with outdoor temperatures in February ranging between +5°C (highest temperature measured in February 2013 in Whitehorse) and −47°C (lowest temperature measured in February 2015 in Dawson City). Additionally, the extremely high air humidity (up to 100% as measured in February 2013 in Whitehorse) contributes to the possible onset of frostbite, which, along with other (medical) concerns, may lead to immediate disqualification. Importantly, the weather conditions between editions were not significantly different (Figure 1), detailed information on weather conditions can be assessed in respective weather archives^2^.

Notably, participants walked between 12 and 15 h per day whilst pulling their gear on a sled-like pulk (accounting for 30–40 kg; additionally, participants were allowed up to three drop bags) and, apart from the checkpoints, had to eat, rest and make toiletry arrangements in the outdoor conditions of the Yukon Territory.

More detailed information about the Montane® YAU is provided on the official website of the event^3^.

### Experimental protocol and measurements

**Figure 2:**
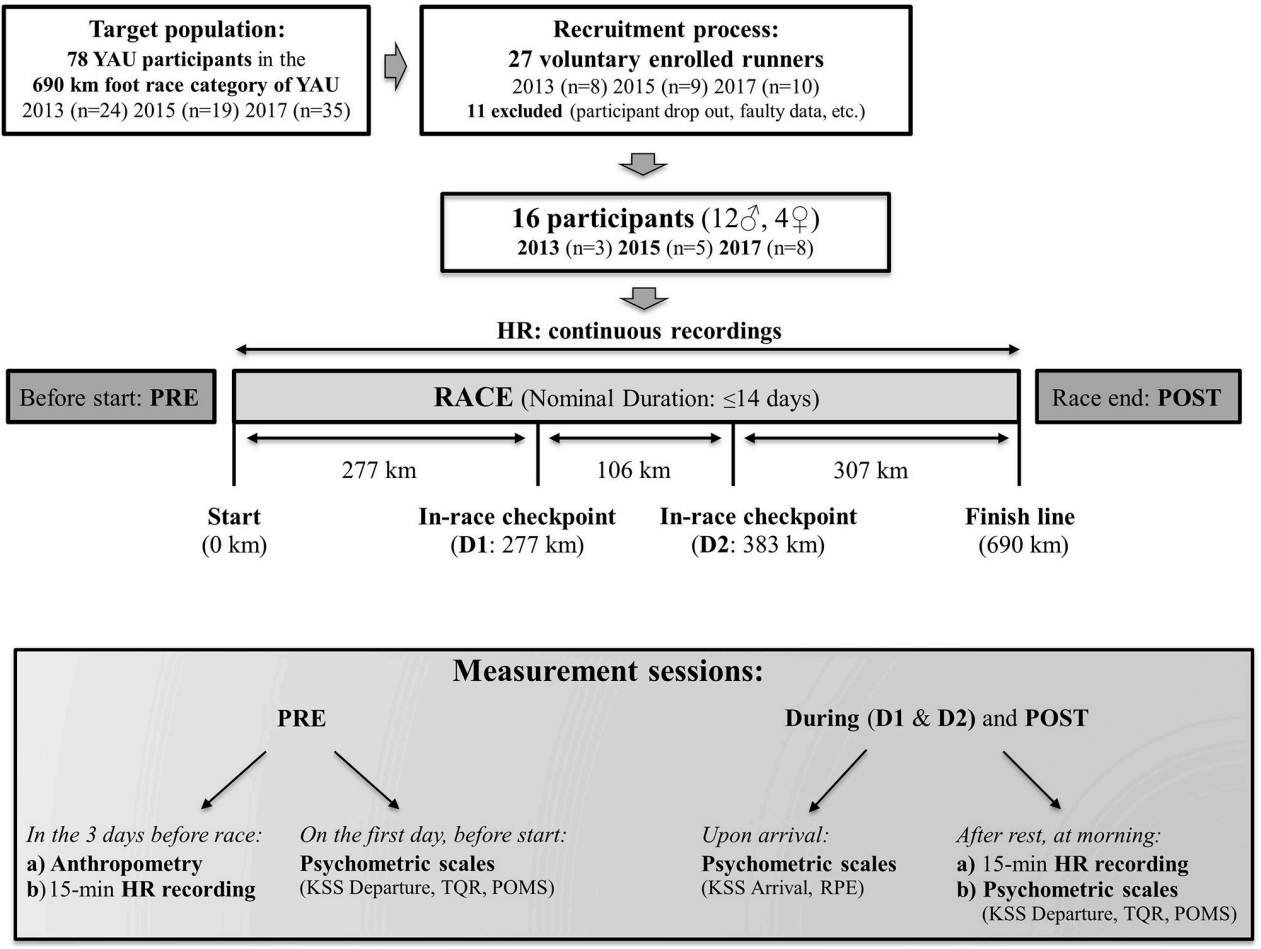
Experimental protocol. Schematic representation of the study protocol and experimental procedure employed in all three YAU races of 2013, 2015, and 2017, except for psychometric assessment (included only in the 2015 and 2017 races).

Experimental protocol details are depicted in Figure 2. At two out of ten in-race checkpoints, we respectively implemented two in-race assessments, so that, in summary, measurements were performed: (1) at baseline during the 3 days preceding the race in Whitehorse (PRE), (2) at the Carmacks in-race checkpoint at 277 km (During 1, D1), (3) at the Pelly Crossing in-race checkpoint at 383 km (During, D2) and (4) immediately after completion of the race in Dawson City at 690 km (POST).

The in-race checkpoints had to be selected for measurement implementation due to essential practical concerns. They had to be indoors facilities buildings with sufficient space, comfortable ambient temperature and low noise in order to perform measurements under controlled conditions, as well available electricity and that it was accessible by car for the investigators. Exemplarily, several of the race checkpoints were mere tents that did not meet these criteria and therefore, the study checkpoints were chosen as they were. Thus, the distance between the race start (i.e., PRE) and the first in-race assessment (D1) accounted for over a third (277 km) of the entire race-course and additionally, in this period, athletes would face the most strenuous weather conditions (which tend to ameliorate toward the second half of the month; see Figure 1). As the second assessment was performed at 383 km (D2), the distance between D1 and D2 (as well as the time to cover it, which accounted for only 30 h in some subjects) was the shortest between the measurements and, in fact, more than 50% less than the other two distances. Conversely, the distance between D2 and POST was again very high (307 km) and additionally, the terrain in the last third of the course accounted for the greatest elevation gains (see section The Yukon Arctic Ultra: The Longest, the Coldest Ultramarathon).

#### Baseline assessment

During the three race-preceding days (PRE), baseline anthropometric data (age, weight and height) were obtained. Weight was measured using a calibrated scale (Seca® GmbH, Hamburg, Germany) and height was taken from the participants' interview.

Fifteen minutes baseline recordings of beat-to-beat HR to assess HRV were collected with a HR monitor (RS800CX Polar Electro Oy, Kempele, Finland), which is widely used and validated for HRV assessment (Wallén et al., 2012). HR recordings were performed in supine position upon awakening (between 5 and 10 a.m.) directly after participants had slept 6–8 h the previous night. The athletes had not consumed food, beverages or stimulants (e.g., coffee) in the 2 h before the recording and were instructed to breathe normally, avoid speaking and moving during the data collection. Additionally, it was ensured by the investigators that subjects would not fall asleep. With the limitations of this specific in-field study, special attention was devoted to performing data collection sessions in a quiet and comfortable setting, with participants lying in a bed or on a sleeping sleeping mattress, ambient temperatures between 17 and 23°C, and reduced light.

On the morning of the race start before departure, additionally, psychometric assessment was performed (see Figure 2 and section Psychometric Assessment).

#### In-race and POST assessment

Upon arrival at the in-race checkpoints, as well as at the race finish, psychometric scales were administered according to physiological needs and conditions of arriving competitors. Participants then had a few hours of rest (ranging from 4 up to 6–8 h) and upon awakening (at morning, between 5 and 10 a.m.), HR data was collected, as at baseline, to assess HRV. Afterwards, before departure, psychometric assessment again took place (see Figure 2 and section Psychometric Assessment).

Indoors ambient conditions between the different measurement facilities were comparable, with special attention dedicated to a quiet and comfortably warm setting with reduced light exposure.

Moreover, throughout the entire race, participants were continuously (day and night) monitored by means of a heart rate monitor (RS800CX Polar Electro Oy, Kempele, Finland - sample rate 15 s).

### Data analysis

#### Performance assessment and heart rate continuous recordings

The official time at the end of the race for each participant who reached the finish line was collected, together with the times and the respectively completed distance for each participant who had to withdraw. Subsequently, the mean running velocity of the race was calculated from the total recorded time and the total distance covered [*time* (*h*)/*space* (*km*) = *velocity* (*km/h*)]. By collection of in- and out-going times at in-race measurement points for each participant, both split times and velocities could be computed, allowing detailed assessment of performance. Additionally, continuous HR measurements served to determine exercise intensity, as well as resting quality (in respect to HR expressed as a percentage of calculated maximal HR). The continuous HR recordings, collected during the race, were screened for quality (no more than 3% signal lost/disturbed). The average, maximum and minimum HR were determined per each selected race period, and the values were normalized with respect to the individual age-related maximal HR (HR_max_) (Tanaka et al., 2001). This provided further information about exercise intensity and quality of rest. Specifically, data were divided into four time-segments, according to the selected period of recording: (i) HR recorded in the first 36 h following the race start (D1a), (ii) during the 24 h before arriving at D1 (D1b), (iii) during the 24 h before arriving at D2 (D2a), and (iv) HR recorded during the last 24 h before finishing the race (D2b). Collected HR data were then expressed as a percentage of the HR_max_, for average exercise intensity (ExHR) and average resting HR (RestHR). This approach was selected to allow comparison with parameters assessed at checkpoints (i.e., psychometric and HRV analysis) and, by classifying data, served to better interpret findings.

#### Heart rate variability assessment

An expert operator visually inspected the R–R interval series, and with the support of a dedicated software (Kubios HRV ver. 2.1, Kuopio, Finland), premature beats or artifacts were removed. The filter threshold was set at the “low” level (Tarvainen et al., 2014) and only files 15-min long and including less than 0.3% of beats recognized as artifacts were considered; then the last 10 min were selected for HRV assessment, to better standardize the analysis. After providing the normal-to-normal (NN) interval series, HRV was assessed as validated indices of autonomic cardiac modulation, based on time-domain, frequency-domain, and complexity (European Society of Cardiology and the North American Society of Pacing and Electrophysiology, 1996). Specifically, as for time-domain analysis, the root mean square of the successive RR differences (RMSSD), an indirect index of vagal activity, was calculated. Furthermore, NN50 statistics were computed, specifically, the hourly number of increases (NN50+) or decreases (NN50–) between consecutive NN intervals larger than 50 ms (Ewing et al., 1984), as well as the percentage of such differences with respect to the total number of NN intervals (pNN50+ and pNN50–) (Bigger et al., 1988; Merati et al., 2015). The NN50 statistics may reflect the rate of “vagal bursts,” as bursts of vagal outflow are producing NN intervals greater than 50 ms (Ewing et al., 1984). In the frequency domain, the total spectral power density (TP) was assessed together with its components: (i) high frequency (HF) band (0.15–0.40 Hz), which depends mainly on parasympathetic activity and is synchronous with the respiratory sinus arrhythmia; (ii) low frequency (LF) band (0.04–0.15 Hz), which depends on both parasympathetic and sympathetic activity; and (iii) LF/HF ratio, which is currently considered a marker of sympathovagal balance (Ewing et al., 1984). In the non-linear domain, as for complexity analysis, the following indices were assessed: (i) the HR sample entropy (SampEn), which measures the level of irregularity of the NN interval series and mirrors vagal activations or sympathetic deactivations (Porta et al., 2008); (ii) the short-term self-similarity coefficient (α1) and long-term self-similarity coefficient (α2) of NN intervals, as assessed by detrended fluctuation analysis (DFA), mentioned here respectively as DFAα1 and DFAα2 (Peng et al., 1995). Both indices may be affected by parasympathetic tone, whereas, for example, higher DFAα1 is associated with sympathovagal balance increase or vagal tone decrease (Penttilä et al., 2003). The significance of DFAα2 has not yet been completely elucidated, as there is indeed only scarce evidence within the literature. However, it seems to be associated with alertness (Ivanov et al., 1999) and may be influenced by sleep stages, being higher in awake states and REM sleep with respect to light and deep sleep (Schumann et al., 2010).

All HRV indices, except for NN50 statistics (manually calculated), were assessed by means of the Kubios HRV software, ver. 2.1 (Kuopio, Finland), a free available software to assess HRV, widely used in the scientific literature, especially in the field of sport sciences (Tarvainen et al., 2014).

#### Psychometric assessment

##### Karolinska Sleepiness Scale

The Karolinska Sleepiness Scale (KSS) (Kaida et al., 2006), which has been highly validated to sensitively depict objective sleepiness (Kaida et al., 2006; Sallinen et al., 2008), was administered both after rest (before departure in the morning: KSS Departure) and upon arrival (KSS Arrival) (see Figure 2). The athletes were asked to rate their subjective sleepiness on a numerical scale ranging from 1 to 10. Specifically, 1–6 are assigned to an “active state” of alertness (1 corresponding to “extremely alert” and 6 to “some signs of sleepiness”) and 7–10 to a “sleepy state” (7 corresponding to “sleepy, but no difficulty remaining awake” and 10 to “falling asleep all the time”).

##### Borg Scales

After rest (before in-race departure and at the finish), subjects were administered the Borg Total Quality of Recovery (TQR) questionnaire (Kenttä and Hassmén, 1998). The Borg TQR has been demonstrated to sensitively represent the individual recovery status (Freitas et al., 2014), whereas the use of recovery and wellness indicators has exhibited important validity in the monitoring of athletes (Buchheit, 2015; Bisschoff et al., 2016). It consists of a 6–20 numerical scale, with 6 being equivalent to “very, very poor recovery” and 20 to “very, very good recovery,” so that the obtained score allows determination of the athlete's subjectively evaluated quality of recovery.

Upon arrival at checkpoints or the finish (Figure 2), participants were administered the Borg Rating of Perceived Exertion (RPE) scale (Borg, 1982; Scherr et al., 2013), which is commonly used in athletes to monitor exertion and also the current subjective workload, additionally, in association with cardiac autonomic regulation (Parry et al., 2011; Thorpe et al., 2016; Siegl et al., 2017) and performance (Suzuki et al., 2006). It again consists of a 6–20 numerical scale, 6 being “very, very light” and 20 “very, very hard,” the individual score indicating the athlete's degree of subjectively perceived exertion.

##### Profile of Mood States

At morning, after rest, mood states in the YAU participants were investigated through the Profile of Mood States questionnaire in the short-form (POMS-SF, here referred to as POMS) (Curran et al., 1995). This extensively validated tool is commonly used in athletic monitoring (Hedelin et al., 2000; Leti and Bricout, 2013; Bisschoff et al., 2016) and has been variously observed to be associated with both HRV and performance (Hedelin et al., 2000; Leti and Bricout, 2013; Comotto et al., 2015; Bisschoff et al., 2016). The POMS required participants to state the extent of emotions currently experienced during the last hours (respectively operationalized as “not at all” providing a subscore of 1; up to “extremely,” providing a subscore of 5). Analysis of individual subscores in emotional subcategories subsequently provided an individual raw score representing the 6 main mood states Depression, Vigor, Fatigue, Tension, Confusion and Anger, as well as a total sum score of mood disturbance (POMS Total).

The psychometric assessment of mood states in our subjects was further analyzed by comparison with normative data for an athletic sample (Terry and Lane, 2000). This data had been obtained in mixed general athletic samples as well as, amongst others, subgroups of athletes at different competition levels and situations (pre- or post-competition, etc.) (Terry and Lane, 2000). In accordance with Terry, raw scores were transformed to a normalized T-Score (using the individual raw score, group mean and group standard deviation) through the formula: *T-Score* = *50* + *[10* + *(raw score* − *group mean)]/SD*. This transformation converted raw scores to normalized scores on a standard scale with a mean of 50 and a standard deviation of 10, so that individual results could be compared with normative sample data. POMS normative scores of athletes from various sport disciplines, plotted against college student norms originally obtained by McNair in 1971 (McNair et al., 1971), show a distinctive pattern of mood states in athletes compared to sedentary populations, which is referred to as the *Iceberg Profile* (Figure **9A**). Specifically, athletes have been found to account for significantly higher Vigor, whereas all other (negative) dimension scores remain below mean values for non-athletes, i.e., “under the surface.” This distinctive profile has been proposed to indicate greater mental health and reduced mood disturbances in athletic subjects compared to sedentary populations. By plotting individually obtained values against normative data, this specific pattern of higher positive mood and mental health in our participants compared to normative data of sedentary subjects could therefore be assessed.

Moreover, by analyzing result scores of the administered psychometric scales, the so-called *psychological wellbeing* (Scully et al., 1998; Johnston et al., 2015; Saw et al., 2016) was evaluated. A higher psychological wellbeing would correspond to an overall low score both for POMS Total (i.e., higher Vigor, lower Fatigue, Tension, Confusion, Anger and Depression scores) and for fatigue scales (i.e., Borg RPE and KSS), and inversely higher scores for Borg TQR.

#### Statistics

Data are reported as means ± standard deviations (m ± SD), if not otherwise stated. From 27 enrolled competitors, we included 16 in the statistical analysis (due to early dropouts before D1, as well as related to HRV-data availability and quality). As a result of the extreme conditions of the competition, several participants withdrew before course completion (see section Performance). Therefore, after the race, the entire sample of all participants (ALL) was divided into two subgroups: finishers (FIN: measurements throughout the race until POST) and non-finishers (NON: measurements until D1) (see Table 1). Normal distribution was tested with Shapiro-Wilk and variance with the Equal-Variance-Test. A log-transformation was applied to frequency domain indices to attain normal distribution (Castiglioni et al., 2011). According to the distribution, the variance of HRV parameters, exercise intensity and rest quality and psychometric measurements over the entire race-course in FIN was tested with one-way repeated measures analysis of variance (One-Way RM ANOVA) and *post-hoc* Student's-Newman-Keuls-Test, or Friedman ANOVA on ranks with *post-hoc* assessment through Tukey's Test (i.e., RMSSD, pNN50+, POMS Depression, Borg TQR). In addition, differences in weather conditions between editions were assessed with One-Way RM ANOVA. As all participants reached the first checkpoint, we could implement a direct comparison between the two groups regarding PRE and D1 by applying two-way repeated measures ANOVA (Two-Way RM ANOVA), after normality was passed. Psychometric assessment of mood states was further analyzed through comparison with normative data for athletic samples. In accordance with Terry (Terry and Lane, 2000), raw values of mood states were conversed to standardized T-Scores. Hence, T-Scores could be plotted against the athletic sample mean in order to assess expression of the *Iceberg Profile*, which represents specific mood profiles in athletic subjects. Unpaired Student's *t*-Test was used on raw values, as well as computed T-Scores, to allow comparison of significant differences between YAU participants and normative data for mixed athletic samples. In order to further analyze mood states in YAU competitors, comparison of baseline values (as individual raw scores) with normative data obtained in athletes directly pre-competition, as well as with normative data obtained in athletes post-competition, was performed by application of unpaired Student's *t*-Test. Correlations between HRV indices and psychometric measurements, as well as correlations between HRV indices or psychometric measurements and performance were assessed with Pearson Product-Moment-Correlation or, if normality was not passed, Spearman Correlation. All statistical analyses were performed using SigmaPlot 12.3 (Systat Software, San José, CA, USA). The significance level was set at *p* < 0.05.

## Results

### Performance

Of the 16 participants included in the statistical analysis (ALL), 10 successfully completed the course (FIN). Due to general fatigue, cardiovascular distress or gastrointestinal problems, as well as injuries (e.g., sprained ankle), 6 withdrew from the competition at earlier points (NON). Baseline anthropometric characteristics of the two subgroups, based on the completion of the race, are presented in Table 1. Details of performance are depicted in Table 2. The official times recorded among FIN ranged between 225 and 312 h. Specifically, analysis of split times displayed that FIN accounted for 82 ± 15 h (velocity 3.6 ± 0.6 km/h) to reach D1, whereas for NON it took 91 ± 17 h (moving at a velocity of 3.2 ± 0.8 km/h). For FIN, 41 ± 6 h were required to reach D2 (velocity 2.7 ± 0.4 km/h) and 125 ± 21 h to reach the finish line (at a velocity of 2.5 ± 0.4 km/h), so that the overall total finish time (i.e., excluding resting time at checkpoints) was 248 ± 36 h (velocity 2.8 ± 0.3 km/h). A positive correlation between the split times PRE-D1 and D2-POST with respect to the finish time was retrieved (*r* 0.83, *p* 0.01 and *r* 0.93, *p* 0.001, respectively). This shows that the participants, who were the fastest both in the first and in the last part of the race, also accounted for the best overall time at the end of the race. Such correlations were not found for the split time between D1 and D2.

**Table 2:** Performance data.

**Group**	**Gender**	**Distance, km mean (S.D.)**	**Finish Time, h mean (S.D.)**	**Velocity, km/h mean (S.D.)**
FIN	Men	690.0 (0.0)	254.6 (21.8)	2.7 (0.2)
	Women	690.0 (0.0)	300.2 (18.8)	2.3 (0.2)
	All	690.0 (0.0)	268.3 (29.7)	2.6 (0.3)
NON	Men	384.3 (107.4)	139.3 (60.0)	3.1 (1.2)
	Women	278.4 (0.0)	92.0 (0.0)	3.0 (0.0)
	All	366.7 (105.4)	131.4 (57.0)	3.1 (1.0)
ALL	Men	350.3 (106.4)	206.6 (71.4)	2.9 (0.8)
	Women	358.3 (124.3)	248.1 (105.2)	2.5 (0.4)
	All	352.3 (106.7)	217.0 (79.3)	2.8 (0.7)

*Performance data of subgroups (FIN and NON), as well as of all participants (ALL). The overall completed distance and total time to cover it are reported, as well as the overall mean velocity (including resting times). No significant differences between groups*.

### Heart rate continuous recordings

A total of 185 recordings fulfilled the criteria for analytic inclusion. They were obtained in 15 participants, as one competitor of the 2017 race belatedly volunteered to participate in the study and could not be equipped with the measuring device anymore. As reported above, recorded HR was used as a marker of exercise intensity, by normalizing absolute values with respect to the calculated HR_max_. A similar approach was used to define the quality of rest during the actual race, i.e., the time spent at checkpoints was excluded. Results of ExHR (mean HR during exercise as percentage of HR_max_) and RestHR (mean HR during rest in between checkpoints, as a percentage of HR_max_), classified according to the 4 segments mentioned above, i.e., D1a (36 h after race start), D1b (24 h before arriving at D1), D2a (24 h before reaching D2), D2b (24 h before arrival at the finishing line), are displayed in Table 3.

**Table 3:** Average exercise intensity and rest quality.

**Group**	***n***	**HR rec. time-point**	**ExHR, % mean (S.D.)**	**RestHR, % mean (S.D.)**
FIN	9	D1a	70.9 (5.3)	47.4 (8.3)
	8	D1b	62.0 (3.9)^*^	38.4 (4.4)^*^
	8	D2a	62.1 (2.6)^*^	40.0 (3.6)^*^
	7	D2b	59.1 (4.4)^*^	37.0 (4.7)^*^
NON	6	D1a	66.9 (5.6)	44.6 (4.9)
	6	D1b	59.1 (5.8)^*^	40.0 (3.6)
ALL	14	D1a	69.5 (5.6)	46.4 (7.2)
	14	D1b	60.7 (4.8)	39.1 (4.0)
	10	D2a	60.9 (3.5)	39.6 (3.7)
	8	D2b	57.0 (7.1)	36.2 (4.9)

Average HR during exercise (ExHR) and at rest (RestHR) across time-points. Data are presented as a percentage of the maximal HR (HRmax) for mean HR during exercise (ExHR) and mean HR during rest periods (RestHR). D1a: HR recorded in the first 36 h; D1b: HR recorded during the 24 h before arriving at D1; D2a: HR recorded during the 24 h before arriving at D2; D2b: HR recorded during the last 24 h before reaching the finish line. Data for the whole sample (ALL) are also reported.

* *p < 0.05 vs. PRE within subgroup (One-Way RM ANOVA)*.

### Heart rate variability

**Figure 3:**
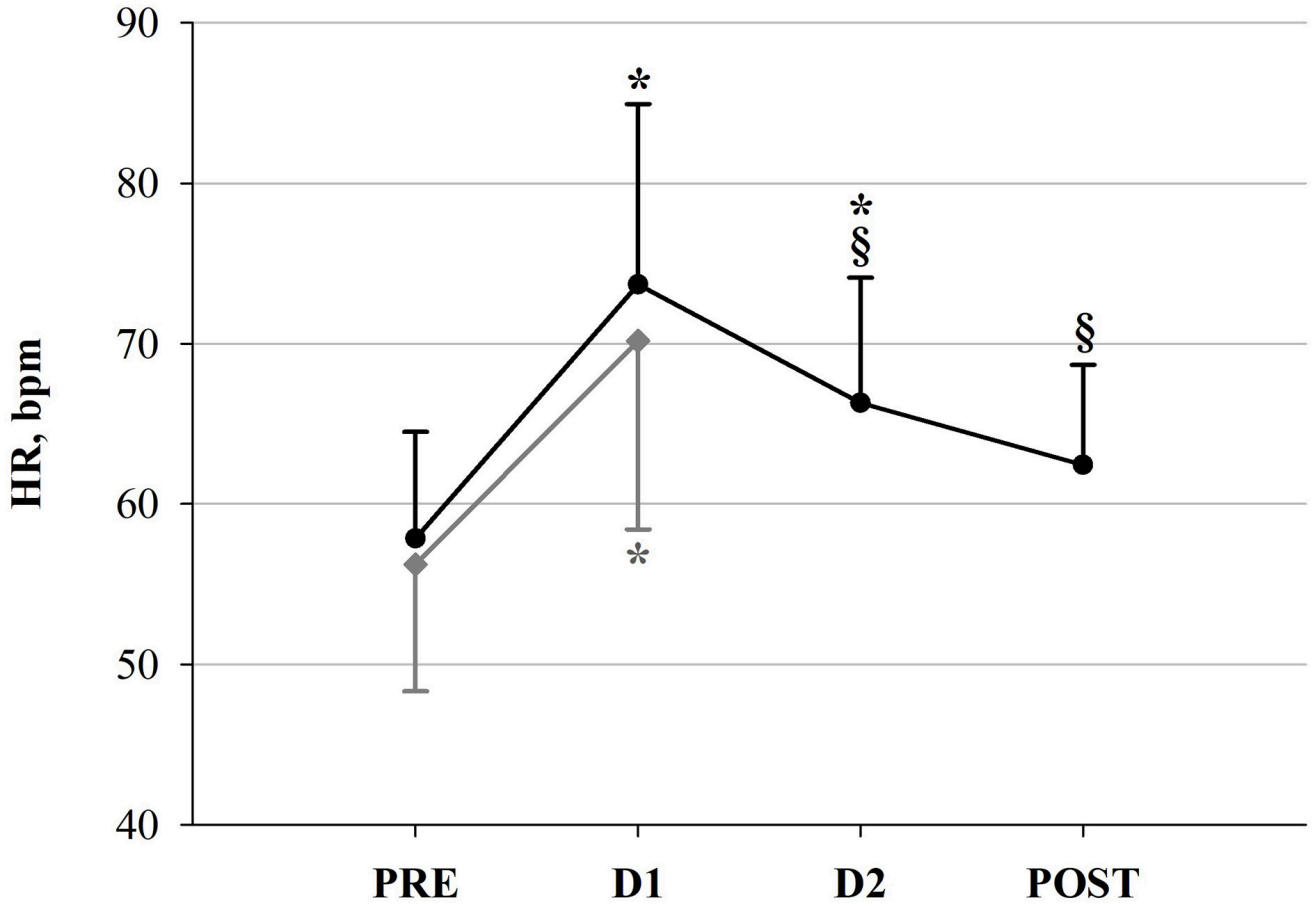
Heart Rate (HR) in FIN (black line) and in NON (gray line) across time-points. ^*^p < 0.05 vs. PRE within subgroup (One-Way RM ANOVA). ^§^*p* < 0.05 vs. D1 (One-Way RM ANOVA).

**Figure 4:**
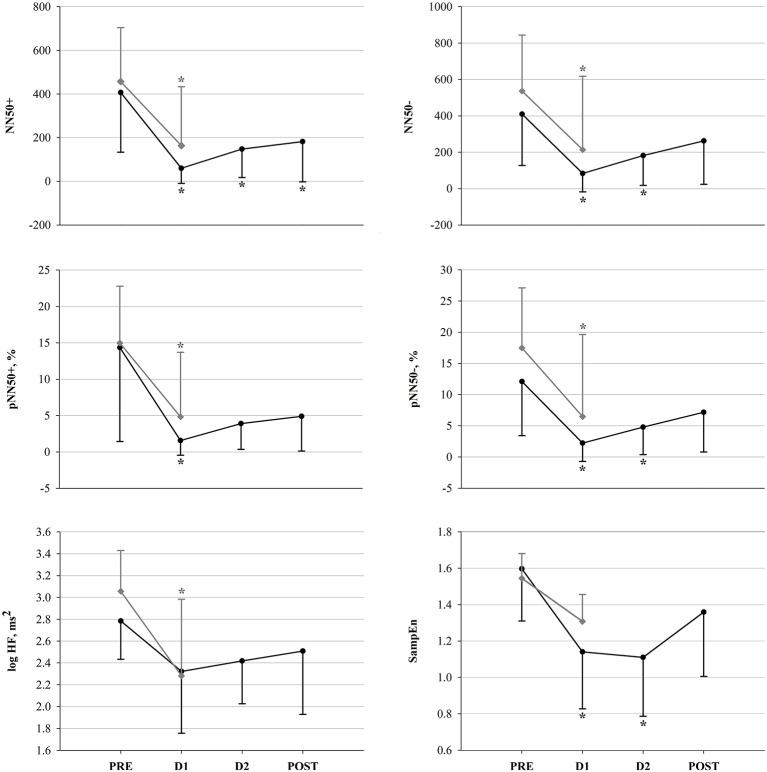
Vagal modulation indices in FIN (black line) and in NON (gray line) across time-points. ^*^p < 0.05 vs. PRE within subgroup (all variables: One-Way RM ANOVA; pNN50+: Friedman ANOVA).

**Figure 5:**
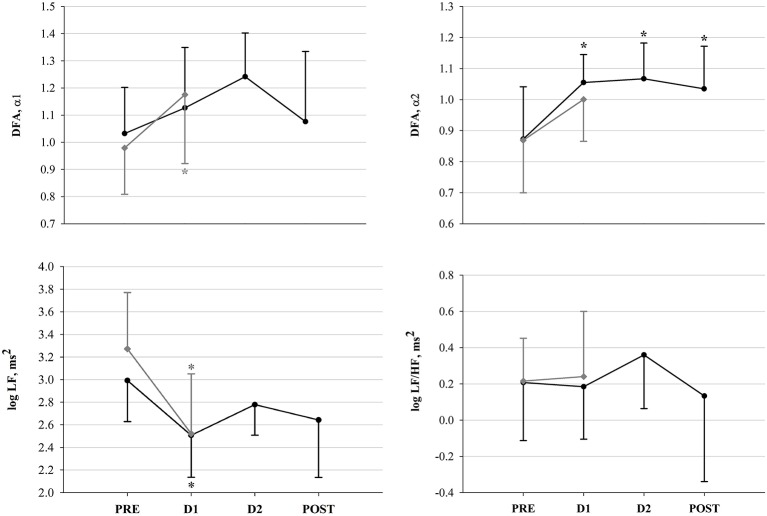
Sympathovagal balance indices in FIN (black line) and in NON (gray line) across time-points. ^*^p < 0.05 vs. PRE within subgroup (One-Way RM ANOVA).

For all 16 participants, a total of 53 R-R interval recordings were available and exhibited sufficient quality for analysis and assessment of HRV. 16 recordings corresponded to PRE, 13 to D1, and 10 to both D2 and POST. Morning HR pre-, post- and in-race (at checkpoints) is depicted in Figure 3 and HRV results are depicted in Figures 4, 5. Figure 4 shows the significant decrease of parasympathetic tone in both groups at D1 compared to PRE, and in the following in-race time-points as for FIN only. Figure 5 depicts sympathovagal balance indices, where a significant decrease at D1 in both groups of log LF lead to no variations of log LF/HF, whereas in FIN a significant increase of DFAα2 across all time-points was retrieved, and in NON only a significant increase at D1 as for DFAα1. Between PRE and D1, an overall decrease in TP could be observed in both groups (FIN: −1,964.7 ms^2^ and NON: −3,699.6 ms^2^), but the decrease was only significant (*p* 0.02) in NON. In fact, in NON the decrease in parasympathetic drive was to some extent greater when compared to FIN, as indicated by DFAα1, which was significantly higher at D1 only in NON (Figure 5), and by the difference between PRE and D1 in values of RMSSD: −34.1 ms in NON (*p* 0.01) and −18.1 ms in FIN (ns) and log HF: −0.8 ms^2^ (*p* 0.04) in NON and −0.5 ms^2^ (ns) in FIN.

**Figure 6:**
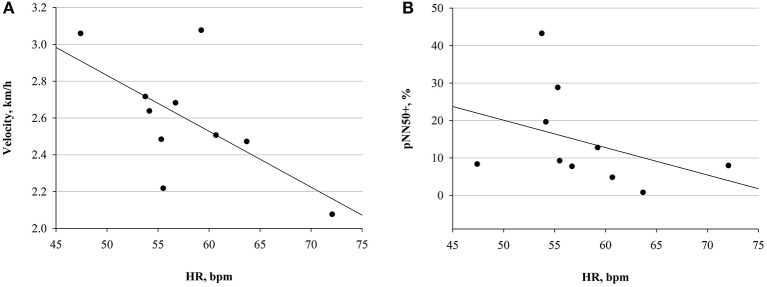
Correlations at PRE in FIN group. **(A)** Correlation between HR and mean velocity (*r* = −0.63; *p* = 0.04). **(B)** Correlation between HR and pNN50+ (*r* = −0.67; *p* = 0.03).

Only in FIN, a significant negative correlation between HR and mean running velocity, as well as between HR and pNN50+, was detected at PRE (Figure 6). The negative correlation between HR and vagal tone indices was observed also at D1, as for pNN50- (*r* −0.82 *p* 0.02), and NN50- (*r* −0.82, *p* 0.02) with respect to HR. This could not be detected in NON.

### Psychometric scales

**Figure 7:**
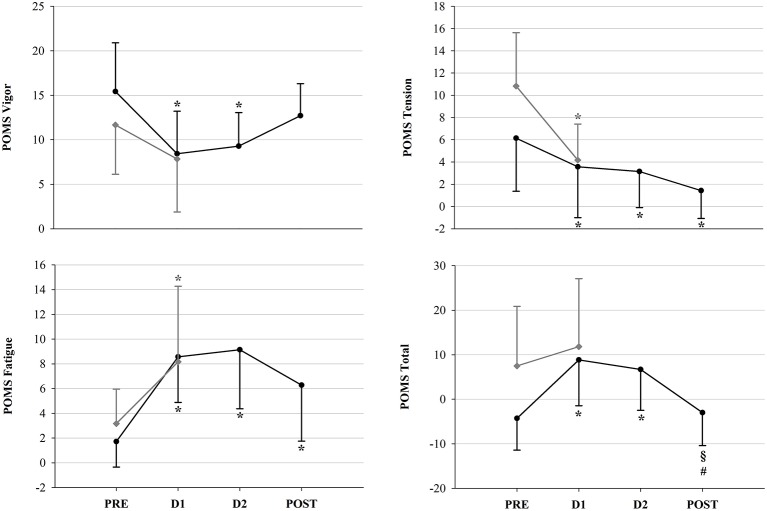
POMS mood state scores in FIN (black line) and in NON (gray line) across time-points: ^*^p < 0.05 vs. PRE within subgroup; ^§^*p* < 0.05 vs. D1; ^#^*p* < 0.05 vs. D2 (One-Way RM ANOVA).

**Figure 8:**
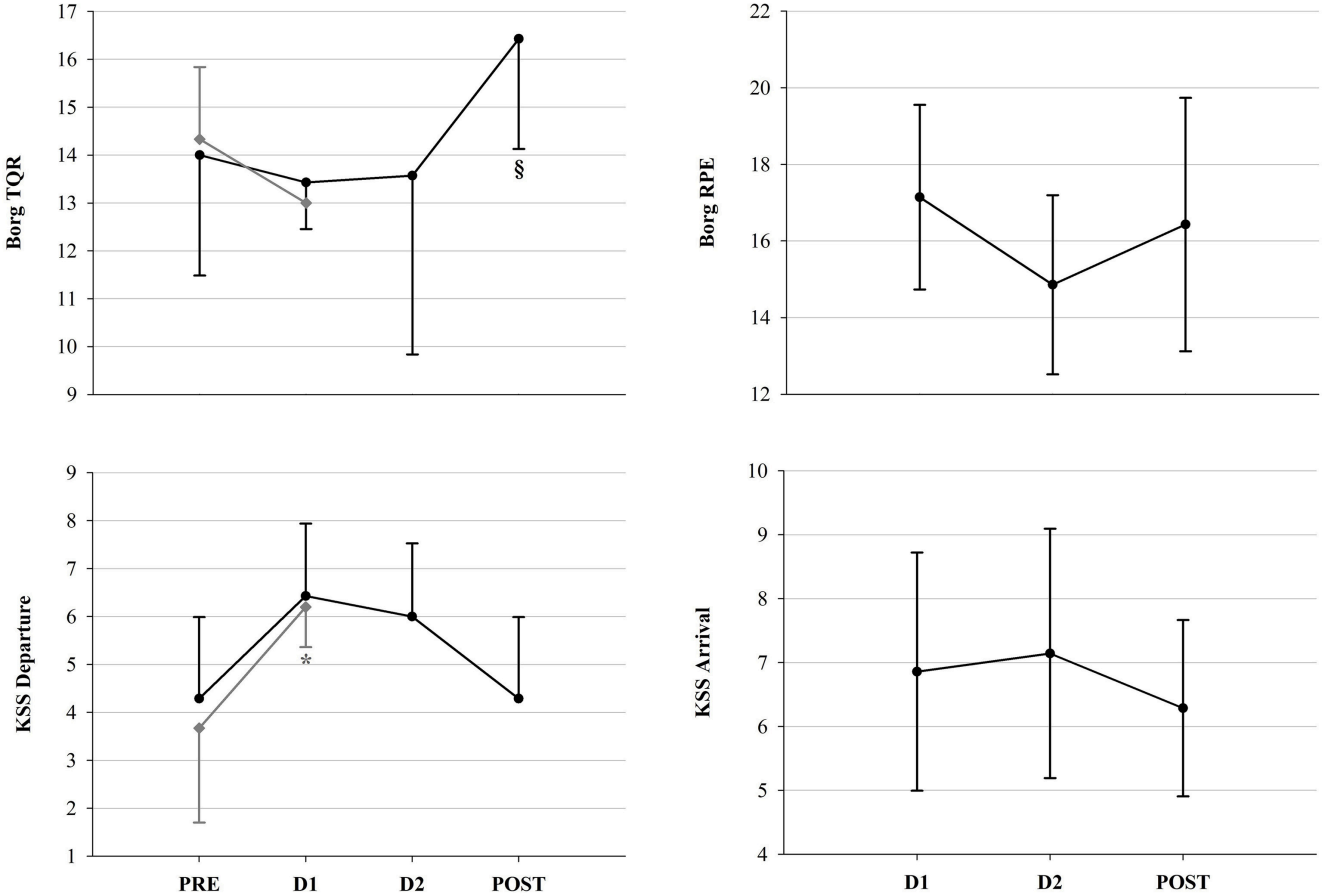
Fatigue scale scores. Borg and KSS scores in FIN (black line) and in NON (gray line) across time-points. ^*^p < 0.05 vs. PRE within subgroup; §*p* < 0.05 vs. D1 (One-Way RM ANOVA).

Psychometric measurements were performed in competitors of the 2015 and 2017 races, so that a total of 45 assessments (13 in PRE, 13 in D1, 10 in D2 and 9 in POST) were included in the statistical analysis. Results of the POMS questionnaire revealed significant decreases in POMS Vigor and Tension, associated with an increase in Fatigue and POMS Total scores (Figure 7). No changes in POMS Depression, Confusion and Anger scores were observed, nor significant differences between FIN and NON at D1. As for fatigue scales, i.e., Borg RPE, Borg TQR and KSS, results are depicted in Figure 8. In NON, values of KSS Departure scores were significantly higher at D1 than at PRE. However, as for the other psychometric scores, no significant between-group differences could be detected.

Nevertheless, at PRE, positive correlations between several indices of vagal tone (RMSSD: *r* 0.86, *p* 0.03; NN50+: *r* 0.90, *p* 0.01; NN50–: *r* 0.89, *p* 0.02; pNN50+: *r* 0.87, *p* 0.03; pNN50–: *r* 0.87, *p* 0.03; log HF: *r* 0.87, *p* 0.03) and Borg TQR were detected in NON only, so the higher the vagal tone, the higher the TQR score. Moreover, at PRE, again in NON only, a negative correlation between POMS Fatigue and pNN50+ (*r* −0.82, *p* 0.04) was observed, which indicates that the lower the vagal tone, the higher POMS Fatigue. On the other hand, in FIN at D1, a negative correlation was observed between HR and KSS Departure (*r* −0.85, *p* 0.02), indicating that the lower the HR, the higher the KSS Departure score; this was associated with a positive correlation between both pNN50– (*r* 0.84, *p* 0.02) and NN50– (*r* 0.84, *p* 0.02) and KSS Departure, which confirmed that a lower HR and a higher vagal drive were coupled with higher sleepiness; additionally in FIN at D1, there was a positive correlation between Borg TQR and DFAα2 (*r* 0.81, *p* 0.03): the higher the TQR score, so the quality of recovery after rest, the higher the DFAα2.

**Figure 9:**
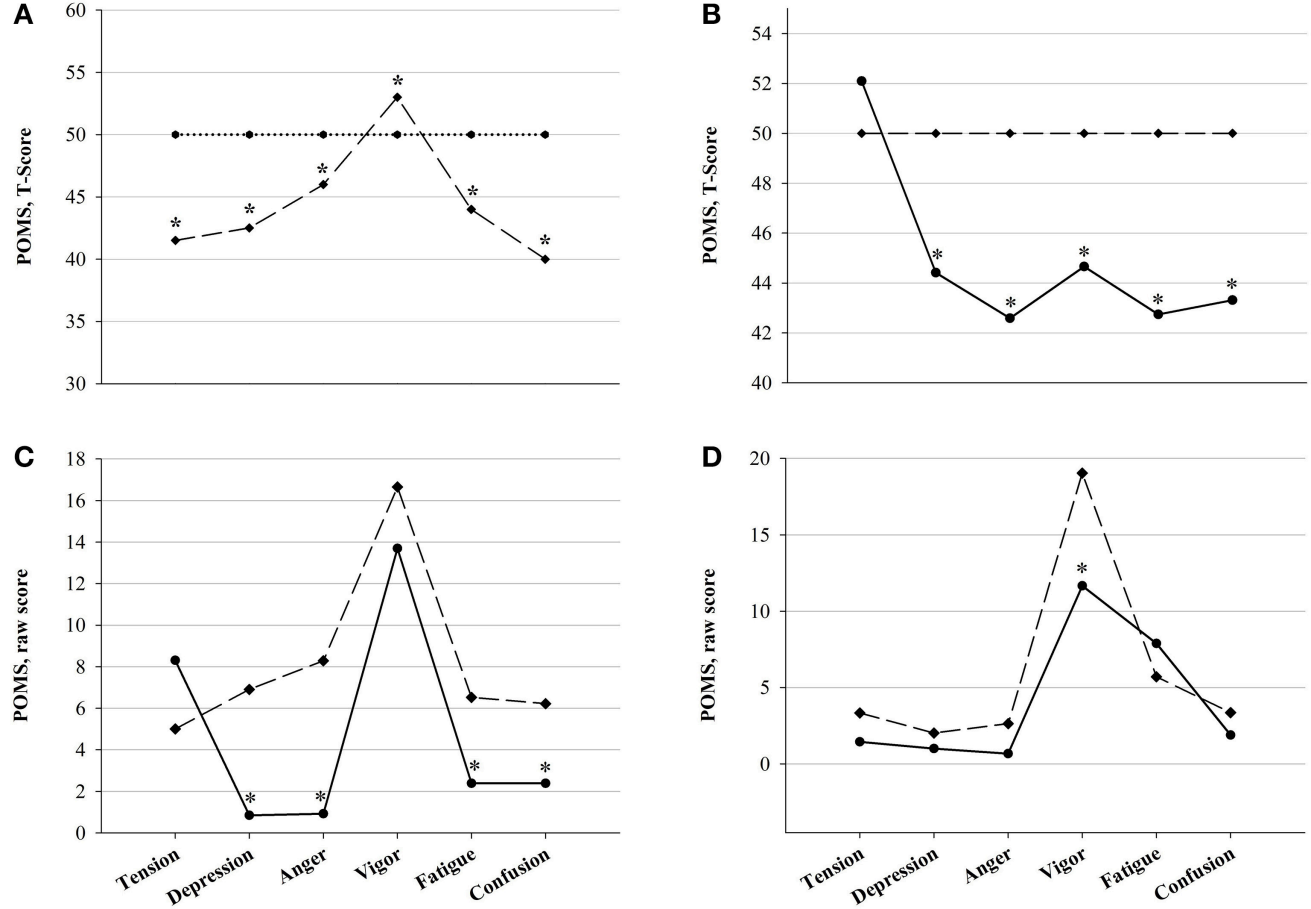
Comparison of POMS results with normative data (Unpaired Student's *t*-Test). **(A)** *Iceberg profile* of mood states in an athletic sample (dashed line) plotted against sedentary subject norms (dotted line), **(B)** Mood states in all YAU subjects (ALL, solid line) plotted against normative data for mixed athletic samples (dashed line), **(C)** Mood states in ALL at PRE (solid line) plotted against normative data for pre-competition assessment (dashed line), **(D)** Mood states in ALL at POST (solid line) plotted against normative data for post-competition assessment (dashed line): ^*^p < 0.05 between groups.

POMS T-Scores of YAU participants were plotted against normative data to provide the above-mentioned *Iceberg Profile* (Terry and Lane, 2000), which depicts POMS Vigor to be significantly higher and all other (negative) dimension scores to be significantly lower compared to mean values for a sedentary population. Analysis of variance between YAU participants and normative athletic data revealed distinctive differences, which are depicted in detail in Figure 9B. In comparison to the athletic sample, raw and T-Scores at baseline were significantly lower in ALL regarding POMS Depression, Anger, Fatigue and Confusion, but also Vigor. This was similarly observed when plotting YAU scores against normative data for athletes before and after competition. Compared to pre-competition normative data, YAU subjects at PRE displayed significantly lower Depression, Anger, Confusion and Fatigue, whereas Tension and Vigor were not different (Figure 9C). At POST, there were no significant differences between normative data for athletes in post-competition situations and the YAU subjects, except for significantly lower Vigor in YAU (Figure 9D).

## Discussion

To our knowledge, this is the first study investigating cardiac autonomic modulation and psychological correlates during ultramarathon in a subarctic environment. This setting provided the unique combination of three extreme environments: (i) ultra-endurance exercise performance (Perini and Veicsteinas, 2003; Scott et al., 2009), (ii) arduous environmental circumstances (Maughan et al., 2007), such as severe continuous cold exposition, and (iii) sleep deprivation/disturbances, induced by the condition of outdoor living during the race (Stein and Pu, 2012). The interplay of each single component of this *three-folded stress stimulus*, amplifies and affects several physiological and psychological aspects, which may be reflected in physical performance outcomes. Regarding race results, 10 of 16 subjects successfully completed the 690 km ultramarathon. Taking into account characteristics and conditions of the competition (see section The Yukon Arctic Ultra: The Longest, the Coldest Ultramarathon), the distribution of measurements sessions (see section Experimental Protocol and Measurements and Figure 2) holds great importance to interpret observations. In fact, the greatest effect on autonomic cardiac modulation, mood and fatigue was observed in the race segment between PRE and D1 (i.e., more than one third of the entire race); this may have been related to: (i) the initial stress of entering the race (i.e., performance demands coupled with environmental conditions), (ii) the different characteristics of the three parts of the race (see section Experimental Protocol and Measurements), and (iii) the running strategy of successful participants. Indeed, by analyzing the split times between measurement sessions, a positive correlation between the first and the final split time was found. This indicates that the fastest competitors in PRE-D1 (and D2-POST), were also the fastest finishers. The challenge of this first part of the race would be significantly underlined by this observation, and it provided ulterior evidence of the central role of the ability to cope with in-race demands at very early time-points (i.e., directly after entering the race) for optimal adaptation. Therefore, during the first kilometers, successful competitors could already be recognized. This is in line with previous investigations on early recognition of successful competitors by their initial pacing strategies (Renfree et al., 2016; Bossi et al., 2017). Specifically, Lambert et al. observed more successful competitors in a 100 km ultramarathon to display higher velocities than lower performing athletes in the early race stages (Lambert et al., 2004). Moreover, the continuous significant decrease in ExHR in FIN, associated to a concomitant decrease of RestHR (*p* < 0.05 across all time-points vs. PRE), reflected decrements in performance, while the need of rest increased. On the other hand, the continuous decrease in RestHR may also indicate higher quality of recovery (Waldeck and Lambert, 2003; Silvani and Dampney, 2013), demonstrating that successful competitors had higher recovery potential, as they attained a higher quality of rest. Indeed, by comparing FIN and NON, we found a significant decrease of RestHR between PRE and D1 in FIN only.

In line with Millet (Millet and Millet, 2012), our observations show that ultramarathon might be an excellent model to test the adaptive potential under extreme conditions. However, in our case, we have to take into account not only potential effects of ultraendurance exercise, but the interplay of three factors (i.e., *three-folded stress stimulus*) on influencing autonomic cardiac modulation and psychometric aspects: strenuous exercise, living outdoors in subarctic winter and sleep deprivation/disturbances.

### Autonomic cardiac control and endurance

An overall reduction of vagal drive, as well as total HRV, has been widely observed during acute exercise and competition (Perini and Veicsteinas, 2003; Buchheit et al., 2010). After cessation of exercise, parasympathetic predominance is gradually restored, depending on the preceding intensity, the training status and the quality of recovery, as reported also in previous studies on ultramarathon (Gratze et al., 2005; Scott et al., 2009; Foulds et al., 2014), and this is in line with our results. However, due to the very long distance of YAU we did observe already a vagal tone recovery before the end of the race, in successful participants (Figure 4). Considering that our HR recordings were collected after several hours of rest, early at morning during the race, the significant increase in HR (Figure 3) clearly describes the inability for participants to recover completely, as HR remained significantly higher at both D1 and D2. Nevertheless, at D2, HR decreased again with respect to D1 (−7.8 bpm, *p* 0.03), remaining, however, significantly above baseline values. At POST, HR was significantly lower than at D1 (−11.3 bpm, *p* 0.004) and not different from PRE. This would demonstrate that successful competitors (i.e., FIN) were able to positively adapt by recovering toward baseline conditions. This specific trend of HR can also be compared with observations in functional overreaching training interventions, where, initially, the increased training stimulus promotes a decrease in vagal drive (i.e., increase in HR), but, due to optimal adaptation, this is subsequently recovered (Buchheit, 2015; Bellenger et al., 2017). Indeed, in our participants, the increased HR between PRE and D1 was modulated via a significant attenuation in vagal tone in both groups (Figure 4). This, associated with a significant concomitant decrease of log LF, lead to an overall reduced HRV (see result section for TP). Additionally, evidence of reduced parasympathetic drive at D1 was further underlined by significant decreases in SampEn in FIN (Figure 4). In line with previously investigated implications of reduced SampEn values, this suggest lower responsiveness to environmental stimuli under attenuated entropy (Sassi et al., 2015). Interestingly, our findings indicate that in unsuccessful participants (i.e., NON), this parasympathetic drive decrease was to some extent greater than in FIN. This was shown firstly by the significantly higher DFAα1 at D1 in NON only (Figure 5), which has been associated to vagal tone decrease (Penttilä et al., 2003), and secondly by the difference in values of RMSSD and log HF for NON vs. FIN between PRE and D1 (see results section). Taking into account that in athletic subjects, stress has been associated to lower HRV and depressed parasympathetic drive (Nuissier et al., 2007; Cervantes Blásquez et al., 2009), this suggests that successful participants were able to efficiently relax and therefore fall asleep. The stronger decrease of vagal tone in NON, indeed, may indicate that these participants, ultimately unable to complete the race, were characterized by an impaired ability to cope with the in-race demands, already at early points of the competition, which may be reflected by lower quality of recovery (i.e., sleep quality impairment), whereas FIN displayed higher recovery potential. In turn, this supports the hypothesis that in such extreme conditions, vagal tone modulations may mirror the individual's ability to adapt, showing in resilient individuals earlier and efficient increase of parasympathetic tone, after the large initial decrement. Between PRE and D1, we also observed the typical reduction of overall HRV, which normally occurs during exercise, as well as a significant reduction of log LF in both groups, thus leading to non-significant changes in log LF/HF ratio (Figure 5). Only in FIN, DFAα2 was significantly higher at D1 compared to baseline and remained higher at both D2 and POST. As the exact implications of this non-linear HRV index have not been elucidated, this is an interesting finding. Previous investigations have reported that DFAα2 would decrease after the application of clonidine, an imidazoline-derived centrally-acting α2-adrenergic agonist and hypothalamic inductor of hypotension, which affects the overall sympathetic activity by resetting it to a lower setpoint (Castiglioni et al., 2011). Conversely, increased DFAα2 values have been reported in subjects who were awake compared to when asleep, but have also been linked to sleep stages, being higher in awake states and REM sleep than during light and deep sleep (Schumann et al., 2010). These findings allow us to hypothesize a link between increased DFAα2 and hyperarousal or enhanced alertness and vigilance, which in this case would be driven by the sympathetic branch of the vegetative nervous system. As we reported a significant increase in DFAα2 during the race, which persisted up until POST, this interpretation concurs very well with our observations (Figure 5). Higher values of DFAα2 could have been induced by an increased need for vigilance (i.e., sleeping outdoors during the subarctic winter in the Yukon Territory), leading to sleep impairment and/or deprivation, and to a general acute stress response promoting hyperarousal. Furthermore, the negative correlation between HR and velocity, paired with the negative correlation between pNN50+ and HR at PRE only in FIN (Figure 6), indicated that a lower HR in association with a higher vagal tone would predict a better performance. In FIN, the persistency of the above-mentioned negative correlation of vagal indices with HR at D1 (see Results section), demonstrated how, in our study, the observed increase in HR was specifically driven by a decrease of parasympathetic tone. This mechanism was mirrored mainly by time domain indices of vagal drive, i.e., NN50 and pNN50 statistics, which are linked to mean HR. Indeed, while pNN50+ quantifies the rate of HR decelerations (increase in successive R–R intervals), NN50– quantifies the rate of HR accelerations (decrease in successive R–R intervals) (Merati et al., 2015). At PRE, our data showed a negative correlation between HR and pNN50+ (i.e., rate of successive HR decelerations) and at D1, HR correlated negatively with both pNN50– and NN50– (i.e., rate of successive HR accelerations). Differences in the distribution of HR decelerations and accelerations have been associated with the enhanced presence of sympathetic modulations, whereas the HR decelerations have been identified as a better marker of vagal activity (Merati et al., 2015). This further demonstrates that at the beginning of the race, between PRE and D1, the reduction of vagal tone determined the increase in HR. Indeed, at D2 and POST, no correlation between vagal indices and HR could be detected. During this second in-between measurement section, HR decreased with respect to D1. Concomitantly, a slight increase of vagal tone was observed (Figures 3, 4). After D1, the correlation between HR and vagal tone disappeared, indicating that the increase of parasympathetic tone was not able to elicit *per se* the decrease in HR. Instead, it would be suggested that the observed HR reduction also occurred due to other concurrent factors as for example psychological states. In fact, at D2, a significant reduction of POMS Total scores (indicating increased negative mood states or disturbance) with respect to D1 was found (Figure 7). This was associated with a decrease of Borg RPE at D2 (although not significant), suggesting that psychological factors were involved in recovering overall wellbeing, and thus were associated with reducing the HR (see section Psychological Wellbeing).

### Psychological wellbeing

We observed an overall decrease of psychological wellbeing across the whole ultramarathon (Figures 7, 8). Interestingly, the POMS Tension item exhibited significantly higher values already at PRE with respect to D1 in both groups, in particular in NON (+6.7 vs. +2.6 in NON vs. FIN). This may reflect pre-competition anxiety. In fact, during the subsequent race, POMS Tension decreased significantly across all time-points. The concurrent increase in Vigor from D2 onwards (as POST values were no longer significantly lower in respect to PRE), may be related to the recovering process of positive mood, but also to the fact that participants were succeeding in the race and the finishing line was getting closer. Between PRE and D1, a significant reduction of positive mood items (lower POMS Vigor and higher Fatigue as well as POMS Total scores) had been observed. Therefore, we can infer that psychometric measurements sensitively reflected the impact of this extremely demanding competition on different subgroups, more strongly affecting those subjects that were unable to cope with the in-race demands. Nevertheless, after D1, it was possible to recognize a particular pattern in FIN, who recovered their wellbeing and positive mood. Indeed, not only POMS Tension scores continuously decreased, but also Vigor again attained values comparable to baseline at POST. Enhanced positive mood or motivation may have furthermore contributed to the observed recovery of vagal tone. In fact, previous investigations have demonstrated associations between enhanced parasympathetic drive in the frequency domain and POMS Vigor, as well as energy index (i.e., the POMS Vigor/Fatigue ratio) (Bisschoff et al., 2016), and the Vigor subscale has been proposed as a marker of the overall autonomic nervous system modulatory activity (Nuissier et al., 2007).

As mentioned above, this finding could be related to the fact that completion of the race was approaching. On the other hand, we found significantly lower POMS Total scores (indicating reduced mood disturbance) paired with higher Borg TQR values at D2 compared to D1 (even if not reaching statistical significance). This reflects a trend of increase in psychological wellbeing. As at POST, POMS Total was similar to PRE values, but significantly higher than at D1 and D2, successful recovery of mood disturbance in FIN is accentuated.

Moreover, during the first part of the race, as mentioned above, the increased HR depicted the inability of participants to recover completely. However, this event was not reported by data of the Borg RPE scale (Figure 8), which, although in-race values had decreased, did not exhibit any significant changes across the race. In this sense, it is likely that in the case of the YAU competitors, the Borg RPE failed to detect the perceived exertion.

Results of Borg TQR in NON showed a significant correlation between vagal indices and TQR scores at PRE, which may suggest that the higher the parasympathetic tone, the higher the perceived quality of recovery, underlining previous findings about the effect of parasympathetic tone on perceived fatigue in athletes (Bisschoff et al., 2016). However, this correlation was not found at D1. Instead, only in NON, KSS Departure scores were higher at PRE compared to D1. As the KSS has been extensively validated to depict objective sleepiness (Kaida et al., 2006), this subjective measurement indicates greater sleepiness, probably due to impaired rest and insufficient recovery in NON compared to FIN.

At PRE, no correlation between psychometric scales and HRV indices was found in FIN. Nonetheless, at D1, KSS Departure correlated negatively with the HR and positively with vagal indices in FIN, i.e., the lower the HR and the higher the vagal tone, the higher the subjective sleepiness upon departure. On the other hand, the concomitant positive correlation between Borg TQR and DFAα2 could suggest that subjects with higher recovery and better sleep quality, were also in a state of enhanced vigilance and alertness, ready to continue on the trail. Nevertheless, we must admit that as we recorded HR early in the morning, just after awakening, and DFAα2 has been reported to be higher in awake states and REM sleep than in light and deep sleep (Schumann et al., 2010), our observations could also be influenced by the circumstances of the measurement sessions, immediately after waking up. The high adaptive potential in our FIN subjects promoting recovery of initially increased mood disturbance, exertion and sleepiness, paired with a concurrently re-increasing subjective recovery status, presents several implications. Possibly, lower sleepiness and therefore higher alertness would yield essential importance for coping with the environmental challenges of the YAU competition. Moreover, sleepiness and fatigue have been associated with impaired cognitive, as well as physical, function and performance (Fullagar et al., 2015). Therefore, the ability to recover from attenuated psychometric wellbeing in our high-achieving FIN once more underlines the importance of adaptability.

Finally, comparison of mood states with normative data for athletic samples (Terry and Lane, 2000) generally displayed lower mood disturbance in our competitors (Figure 9). At baseline, POMS Depression, Fatigue, Confusion and Anger were lower, with Vigor conversely being higher compared to normative scores. Further comparison with normative data for pre- and post-competitive assessment again confirmed the great mental health in our participants, who had significantly higher positive mood than compared to pre-competitive normative values. During the first stages of the race, mood disturbance significantly increased under the exhausting demands, but recovered. Therefore, at POST, mood states in YAU participants (except for Vigor scores) did not significantly differ from normative data in post-competition assessment. To conclude, the high adaptive capacity in our subjects, who attained recuperation of gravely impacted mood states after enduring the extreme in-race conditions and stress stimuli, is again underlined.

Practical applications of these findings are related to training methods, highlighting the importance of high and/or fast increasing vagal tone, and of mood states: the “mind,” i.e., mood state and motivation, plays a crucial role, especially with respect to such a long-lasting and highly demanding competition. In fact, successful competitors were able to perform greatly also in the second part of the race, where the decrease of HR was not coupled directly with higher vagal drive (as instead was in the first part of the race for FIN only), and the intervention of psychological aspects could be hypothesized (see above). All in all, assessment of HRV and psychological profile may contribute to monitor and partly predict performance in such extreme environments.

## Limitations

Given that this is an in-field study in extreme environments, a number of possible limitations must be addressed.

First of all, the sample size of 16 may appear small, however, considering that a total number of only 78 athletes competed in the three investigated editions and 27 of them enrolled in our study, we regard this number to be quite considerable and sufficient under these specific conditions.

Moreover, there is a substantial difference in the distance between the in-race checkpoints (i.e., D1, D2) selected to perform measurement sessions and a study protocol over three equispaced checkpoints may have been favorable. However, the choice of measurement implementation was due to essential practical concerns, as previously mentioned (see section Experimental Protocol and Measurements). These concerns also held essential importance for standardizing as much as possible measurement conditions, (i.e., indoors facility, comfortable setting regarding space, temperature, noise, and light exposure), especially regarding HR beat-to-beat recordings.

Furthermore, we aimed to allow comparison of additional data from HR continuous measurements with HRV and psychometric parameters obtained at measurement points. Therefore, continuous HR recording data were clustered and were split up in the above-mentioned four sections (see section Performance Assessment and Heart Rate Continuous Recordings).

## Conclusion

The main findings of this study are: (i) the extent of the early vagal withdrawal, associated to the timing and potential of its recovery, is crucial for success in this specific competition, (ii) a pre-competition lower resting HR, coupled with a higher vagal tone, would predict a better performance, as already reported in the literature for endurance sports (Gratze et al., 2005; Buchheit, 2014), and (iii) psychological profile and wellbeing is reliably depicted by mood state assessment with the POMS questionnaire, but not by Borg fatigue scales, and again associated with autonomic cardiac modulation. Successful ultramarathoners were coping better already in early stages of the competition, which allowed recovery of cardiac autonomic balance and positive mood, thus associated with higher athletic achievement. Therefore, assessment of HRV and psychological profile may contribute to monitor and partly predict performance in such extreme long-duration competitions in extremely cold environments.

## Author contributions

LR and MM contributed equally to the study by writing the manuscript and analyzing the data. MS designed, planned and implemented the study, secured funding sources, and performed measurements and data collection. AS assisted with the measurements and data collection. AR-R, LR, and MM performed statistical analyses. RC and H-CG contributed to the study design, provided expertise and feedback. LR formatted, and, with assistance of MM and MS, revised the manuscript.

### Conflict of interest statement

The authors declare that the research was conducted in the absence of any commercial or financial relationships that could be construed as a potential conflict of interest.

## Footnotes

^1^https://www.wma.net/policies-post/wma-declaration-of-helsinki-ethical-principles-for-medical-research-involving-human-subjects/

^2^http://climate.weather.gc.ca/historical_data/search_historic_data_e.html.

^3^http://arcticultra.de/en.

^4^Data taken from https://www.timeanddate.com/weather/canada/whitehorse/historic, https://www.timeanddate.com/weather/canada/dawson-city/historic and http://climate.weather.gc.ca/historical_data/search_historic_data_e.html (last accessed December 13, 2017).
